# Supplementary material for: Association between oral microbiome and seven types of cancers in East Asian population: a two-sample Mendelian randomization analysis
Source: Front Mol Biosci. 2023 Nov 21;10:1327893. doi: 10.3389/fmolb.2023.1327893 (PMC10702768; doi:10.3389/fmolb.2023.1327893)
Supplement: Supplementary file 1 [file DataSheet1.ZIP › Supplementary Materials/MR plots for tongue/tongue═╝/Ovarian cancer/pheno.965_to_ovarian cancer_forest.pdf]

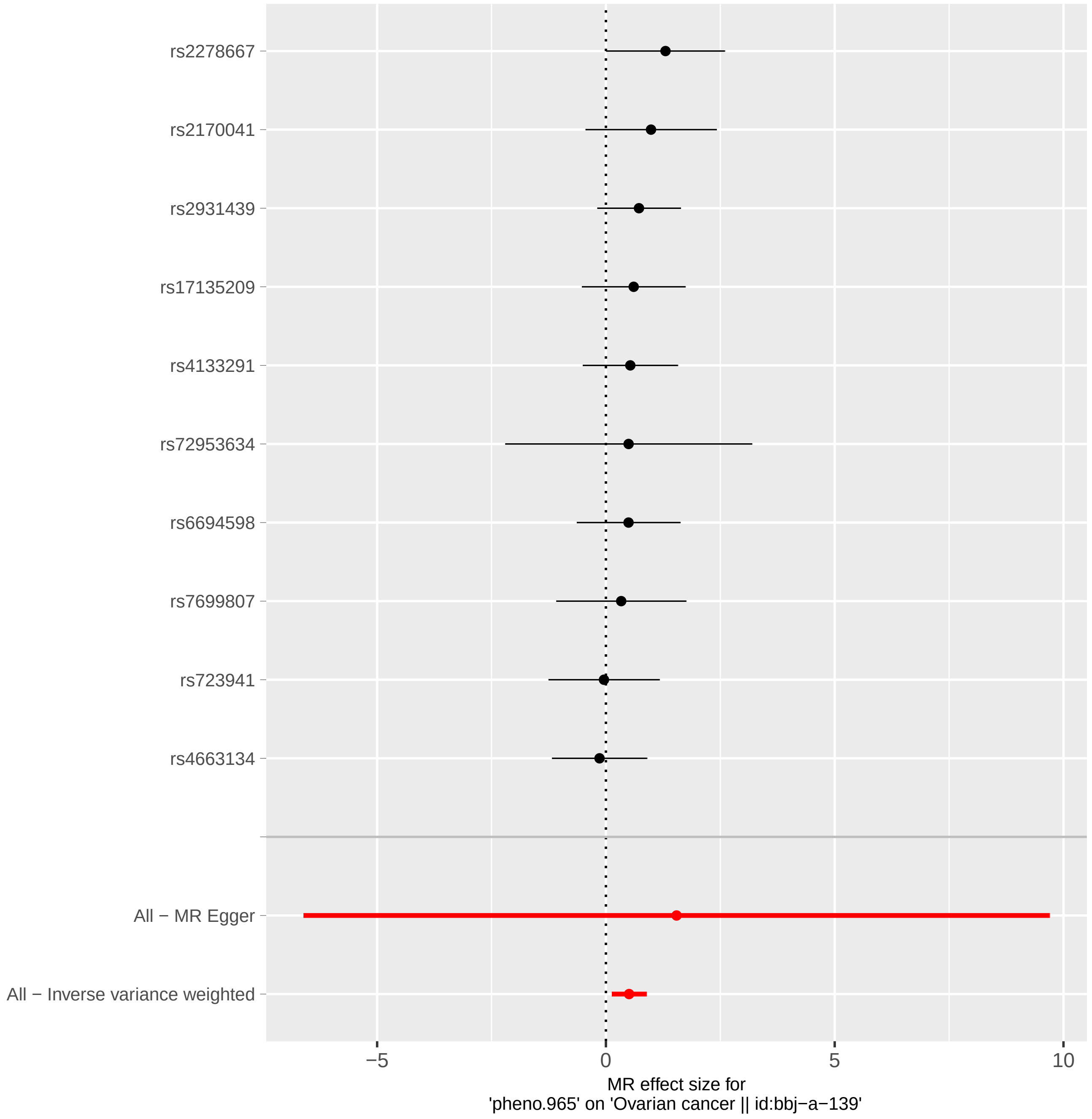

rs2278667
rs2170041
rs2931439
rs17135209
rs4133291
rs72953634
rs6694598
rs7699807
rs723941
rs4663134
All − MR Egger
All − Inverse variance weighted
−5
0
5
10
MR effect size for
'pheno.965' on 'Ovarian cancer || id:bbj−a−139'
